# An agent-based model clarifies the importance of functional and developmental integration in shaping brain evolution

**DOI:** 10.1186/s12915-021-01024-1

**Published:** 2021-05-10

**Authors:** Shahar Avin, Adrian Currie, Stephen H. Montgomery

**Affiliations:** 1 Centre for the Study of Existential Risk, University of Cambridge, Cambridge, UK; 2 Department of Sociology, Philosophy and Anthropology, University of Exeter, Exeter, UK; 3 School of Biological Sciences, University of Bristol, Bristol, UK

**Keywords:** Brain structure, Brain size, Constraint, Concerted evolution, Mosaic evolution, Neuro-evo-devo

## Abstract

**Background:**

Vertebrate brain structure is characterised not only by relative consistency in scaling between components, but also by many examples of divergence from these general trends.. Alternative hypotheses explain these patterns by emphasising either ‘external’ processes, such as coordinated or divergent selection, or ‘internal’ processes, like developmental coupling among brain regions. Although these hypotheses are not mutually exclusive, there is little agreement over their relative importance across time or how that importance may vary across evolutionary contexts.

**Results:**

We introduce an agent-based model to simulate brain evolution in a ‘bare-bones’ system and examine dependencies between variables shaping brain evolution. We show that ‘concerted’ patterns of brain evolution do not, in themselves, provide evidence for developmental coupling, despite these terms often being treated as synonymous in the literature. Instead, concerted evolution can reflect either functional or developmental integration. Our model further allows us to clarify conditions under which such developmental coupling, or uncoupling, is potentially adaptive, revealing support for the maintenance of both mechanisms in neural evolution. Critically, we illustrate how the probability of deviation from concerted evolution depends on the cost/benefit ratio of neural tissue, which increases when overall brain size is itself under constraint.

**Conclusions:**

We conclude that both developmentally coupled and uncoupled brain architectures can provide adaptive mechanisms, depending on the distribution of selection across brain structures, life history and costs of neural tissue. However, when constraints also act on overall brain size, heterogeneity in selection across brain structures will favour region specific, or mosaic, evolution. Regardless, the respective advantages of developmentally coupled and uncoupled brain architectures mean that both may persist in fluctuating environments. This implies that developmental coupling is unlikely to be a persistent constraint, but could evolve as an adaptive outcome to selection to maintain functional integration.

## Background

How are macro-evolutionary patterns in vertebrate brain structure best characterised, and what processes drive those patterns? Answering such questions requires understanding how developmental mechanisms or architectural constraints, as well as selection acting on neural traits, together shape and support behavioural and cognitive evolution. Debates over these conflicting pressures on variation have dominated vertebrate evolutionary neurobiology for decades, with no unified theoretical framework in sight.

At the heart of this debate are two views of vertebrate brain evolution which, at their most polarised, make seemingly opposing predictions while both appearing logically sound and empirically supported. Under one hypothesis, brain components are developmentally coupled such that the size of each component is largely determined by common developmental mechanisms, such as the schedule, timing and duration of neurogenesis [1–3]. This would lead to the majority of brain structures evolving in a ‘concerted’ manner, with the size of separate components being closely predicted by overall brain size [1–4]. Initially, this coupling was discussed as a potential evolutionary constraint, associated with ‘spandrels’ whereby late developing brain regions such as the neocortex, may have expanded disproportionately as a by-product of architectural constraints, before later being co-opted functionally [2]. Proponents of this hypothesis now largely argue that developmental coupling is a mechanism that evolves in response to selection favouring conservative scaling, and is, as such, a potential adaptive mechanism rather than a constraint per se [1, 3]. However, the view that concertedness in itself indicates developmental constraint remains widespread in the literature (e.g. [4–12]).

A contrasting hypothesis instead argues that variation in brain components is largely developmentally independent of both other brain structures, and of total brain size, allowing them to respond to targeted selection pressures in a ‘mosaic’ way [13–17]. Mosaic evolution is often discussed as facilitating neural adaptations, reflected in non-allometric changes in brain structure, but it also invokes stabilising selection to otherwise maintain scaling relationships between co-evolving, functionally interdependent brain components [14, 18]. In essence, the mosaic model favours ‘external’ explanations that emphasise the role of both divergent and coordinated selection in driving both independent phenotypic evolution *and* co-variation of brain structures, while ‘concerted’ theorists stress ‘internal’ mechanisms which emphasise the role of developmental coupling as a route to maintaining scaling relationships [19].

Perhaps confusingly, both hypotheses have at times been supported by analyses of the same volumetric data (e.g. [2, 14, 20]). Proponents of the ‘concerted’ view of brain evolution pointed to consistent allometric scaling between brain components and total brain size as evidence of strong developmental integration across brain structures [1, 2, 4]. Proponents of the ‘mosaic’ model instead pointed towards co-evolution between brain components that is independent of total brain size, and evidence for ‘grade shifts’ that indicate deviations in scaling between taxonomic groups, as evidence that brain components are caught between distinct selection pressures, and constrained from functional interdependence [14]. Distinctions between these hypotheses have become more nuanced, with the concerted hypothesis incorporating periodic restructuring of the brain, accommodating some mosaic change [3, 21]. But, regardless, universally satisfactory tests of the generality of these hypotheses have proven elusive, there is frequent confusion in the literature between the distinction between patterns and mechanisms of brain evolution, and little data exists on when one mechanism may be favoured over another.

There are two key reasons for this deadlock. First, proponents of concerted and mosaic models are engaged in a ‘relative significance debate’ [22]. Both sides agree that brain evolution exhibits features associated with both concerted and mosaic evolution, but disagree on which pattern dominates across evolutionary time, and why (see for example, [1], p. 299). Relative significance makes hypothesis testing difficult. Neither hypothesis is subject to critical tests, as both accommodate—and even expect—different degrees of departure from the ‘norm’. Alternative views of brain evolution therefore run the risk of being too indeterminate for definitive testing.

Second, tests of these hypotheses are underdetermined by available evidence. Although proponents of both mechanisms can point to support from developmental data (reviewed in [18, 23])—showing, for example, how concerted patterns of brain evolution can be produced by changes in the regulation of neural progenitor cell proliferation [24–27], or how changes in the allocation, rate or duration of cell division among the cell populations that lead to specific regions can produce mosaic changes in brain structure [28–31]—these data are naturally less readily available than volumetric data, and therefore, tests of generalisation are limited. As such, empirical support for concerted or mosaic evolution is most often drawn from comparative analyses of volumetric brain data. These data reflect the outcome of the interaction between competing adaptive and constraining factors and do not, in themselves, provide evidence of the developmental mechanisms involved [32, 33]. This is a critical point, as ‘concertedness’ has frequently become a byword for developmental constraint (e.g. [4–12, 34]), potentially biasing the interpretation and presentation of many studies. However, the mosaic brain hypothesis also predicts co-variation between interdependent brain regions. If the brain is viewed as a network of interdependent networks, these *functional* constraints could produce consistent scaling relationships across brain components — i.e. concerted evolution — without invoking developmental coupling [18]. As inferred through classic evolutionary theory [35], merely recognising a concerted pattern is insufficient evidence to assess alternative mechanisms, or to support the predominance of either hypothesis.

If patterns of phenotypic variation alone are unsuitable for identifying the mechanisms that underpin allometric scaling, what evidence could? As noted by previous authors, ‘it is not the *phenotypic* correlation that matters, so much as the *genetic* correlation’ [36]. Although brain morphology can be highly plastic, responding to effects of the physical or social environment, which may alter the appearance of how brain structures scale within species (e.g. [37, 38]), the majority of comparative studies interrogating patterns of brain evolution implicitly assume these effects are small relative to heritable variation. Quantitative, intra-specific genetic studies provide a test of this assumption and of the relative strength of genetic correlations between brain size and structure. Several recent quantitative genetics studies have found evidence of substantial genetic independence between brain components [5, 8, 39], a central prediction of the mosaic brain hypothesis (reviewed in [18]). However, these studies typically concern standing genetic variation within populations. The developmental coupling hypothesis can accommodate this evidence if much of this genetic variation is mildly deleterious and is maintained in the population due to negative selection being weaker than drift, for example. If this was the case, selection for changes in brain structure or brain size may more frequently act on de novo mutations that are distinct in their developmental effects compared to standing genetic variation, and which are generally purged from the population during times of evolutionary stasis in brain structure, perhaps because they have larger fitness effects. If this were the case, intra-specific studies might not reflect the genetic architecture favoured by selection over evolutionary timescales. Currently, we have insufficient evidence either way. At a comparative level, some authors also argue that both concerted and mosaic patterns are observed in their data, with pairs of structures evolving in a coordinated, or concerted, manner, potentially supported by direct mechanisms linking their development, while others evolve independently [7, 40–42]. This would invoke complex patterns of developmental integration that occur after the major brain divisions are established [40], rather than the more global developmental integration suggested by previous authors, but the limited attempts to test this using molecular data do not currently support this idea [43]. Hence, neither phenotypic data nor currently available genetic data are sufficient to unite views on the relative importance of developmental and functional coupling, constraint and adaptive lability in the evolution of brain structure.

When faced with relative significance and empirical underdetermination, simple mathematical models can help realise basic causal dynamics in a ‘bare-bones’ system and are a way of examining the dependencies between variables that are thought to be important. We can envision ‘bare-bones’ models as tools that serve to make explicit the assumptions and reasoning involved in otherwise linguistic arguments, sometimes revealing previously hidden assumptions [44, 45]. While they lack empirical data, and must therefore be treated with care, they can be critical for informing future empirical studies and aiding the interpretation of existing literature [46]. This is particularly true for relevant significance debates which lack a straightforward way to weigh the importance of multiple mechanisms in different contexts using empirical data. Here, a modelling approach can be used to explore how key variables behave, which can dovetail with existing experimental or comparative studies, or prompt new ones. This approach has recently been applied to debates over the socio-ecological selection pressures shaping brain size [47–50], providing a new approach to the field of evolutionary neurobiology. Here, we introduce an agent-based model of brain structure that allows us to explore the interactions between fitness and constraints derived from selection, development and function (summarised in Additional file 1: Figure S1). In particular, our model allows us to formalise several verbal arguments over the role of developmental coupling in brain evolution; specifically, we ask:

1. Do functional dependencies produce concerted patterns of evolution as well as developmental coupling (e.g. [32, 33])?
2. Can both mechanisms be adaptive (e.g. [1])?
3. Do the costs of neural tissue select against concertedness when selection acts on a specific brain component (e.g. [51])?
4. Is developmental integration evolutionarily labile (e.g. [52]), and do functional dependencies select for developmental coupling (e.g. [32, 52])?
5. Does stabilising selection or constraint on brain size lead to increased frequencies of mosaic evolution (e.g. [51])?

Our model allows us to explore these previously verbal arguments and interpretations of volumetric data. We demonstrate that this ‘bare-bones’ model helps clarify current debates surrounding the evolution of brain structure by capturing the basic evolutionary dynamics at play, and hope that it shifts these debates in a productive theoretical and empirical direction.

## Results

### Do functional dependencies produce concerted patterns of evolution as well as developmental coupling?

**Fig. 1:**
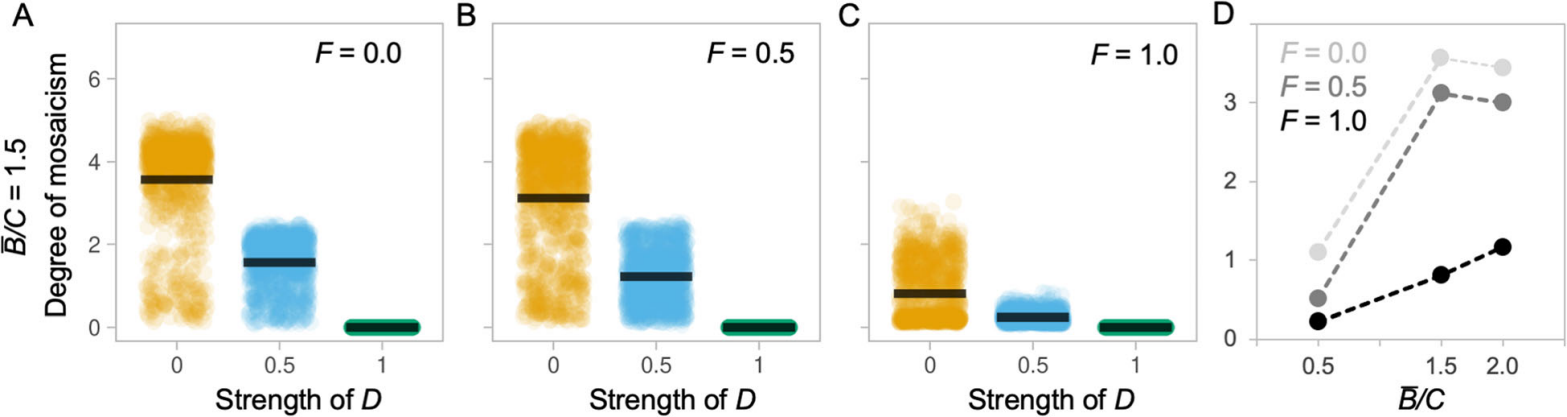
Evolution of ‘mosaicism’ under alternative conditions. **a**–**c** Each plot depicts the ‘degree of mosaicism’ (*y*-axis, defined as the natural log of the ratio between the largest brain component and the smallest brain component in each individual, averaged across the population) as a function of developmental coupling *D* (*x*-axis) at the end of a 100-generation simulation run, compiled over 1000 simulation runs, for a population of 100 individuals with an identical *D*, under different environments, defined by their functional coupling *F*, with an average benefit to cost ratio (*B̅/C*) of 1.5 and a mutation step size of 5%. Each data point is the outcome of one simulation run and the black bar indicates the mean of these runs. **d** Summary of the effects of varying *F* and *B̅/C* on the degree of mosaicism when *D* is 0. See Additional file 1: Figure S2-S4 for full results varying *B̅/C* and *F*, iteration numbers and mutation step size

By varying the degree of both developmental coupling (*D*) and functional interdependence (*F*), between brain components, our model suggests that patterns of ‘concerted’ brain evolution, in which the size of brain components is correlated, can be caused by both developmental and functional coupling (Fig. 1, Additional file 1: Figure S2–4). Unsurprisingly, the probability of patterns of concerted evolution declines with decreasing *D* (*t* = 50.330, *p* < 0.001; Fig. 1d)*.* However, there is a significant interaction between *F* and *D* (*t* = 28.730, *p* < 0.001) whereby, for a given value of *D*, where *D* < 1, the probability of concerted evolution increases with higher values of *F* (Fig. 1a–d), demonstrating that functional interdependence also promotes concertedness. Even when components are completely developmentally independent (*D* is set to 0), high values of *F* can favour comparatively low levels of mosaicism (Fig. 1c). Mutation size also has an effect on the outcome, with more mosaic brains evolving with larger mutation step sizes for a given *D* value (*t* = 133.650, *p* < 0.001; compare Figure S2 and S3). These results illustrate that macro-evolutionary patterns of allometric scaling consistent with concerted evolution are not, in themselves, sufficient to distinguish between alternative mechanistic models of brain evolution.

### Can both mechanisms be adaptive?

**Fig. 2:**
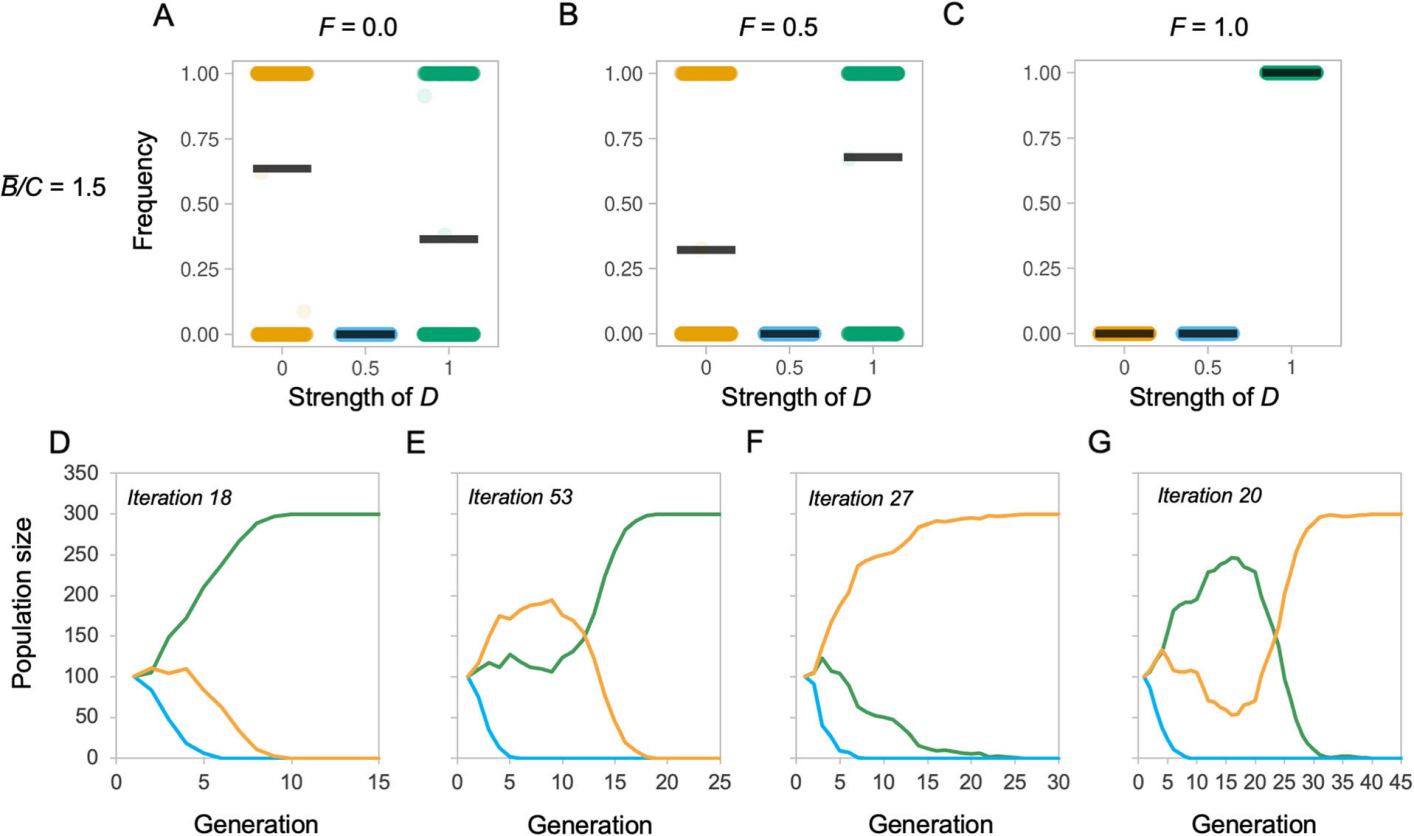
Selected examples of competition between evolving populations with different *D* values. **a**–**c** Each plot depicts the frequency of that *D* value relative to the total population (*y*-axis) as a function of developmental coupling *D* (*x*-axis) at the end of a 100-generation simulation run, compiled over 1000 simulation runs, under alternative environments defined by their functional coupling *F*, with an average benefit to cost ratio (*B̅/C*) of 1.5 and a mutation step size of 5%. Populations are initialised such that there are 100 fully mosaic individuals (*D* = 0.0), 100 partially mosaic individuals (*D* = 0.5) and 100 concerted individuals (*D* = 1.0). Each data point is the outcome of one simulation run and the black bar indicates the mean of these runs. **d**–**g** Selected, representative, individual simulations showing the change in population frequencies over generations for a 5% mutation size and *B̅/C* = 1.5. Colours indicate the *D* value, where yellow is *D* = 0, where blue is *D* = 0.5 and where green is *D* = *1*. These show a general pattern of smooth progression from the starting state of equal populations to one *D* value winning out (**d**, **f**), with only a minority of iterations showing signs of populations ‘swapping’ the lead (**e**, **g**). This consistency is expected under constant selection regimes. See Additional file 1: Figure S6-S8 for full results varying *B̅/C* and *F*, iteration numbers and mutation step size, and Additional file 1: Figure S9–11 for simulations in tailored environments that illustrate parameter effects

By competing alternative *D* values against one another, we found that both *D* (*t* = 21.358, *p* < 0.001) and *F* (*t* = 42.595, *p* < 0.001) affect survival in a particular environment. At an intermediate benefit to cost ratio (*B̅/C* = 1.5), we found that the probability of success of a mosaic, low *D* value population increases when *F* is low, while high values of *F* result in a higher probability of success for the concerted, high *D* value population (Fig. 2a–c; Additional file 1: Figure S6-S8). A ‘partially mosaic’ population (*D =* 0.5) is very rarely favoured (Fig. 2a–c; Additional file 1: Figure S6-S8). These comparisons indicate that variation in selection across components alters the outcome of competition between populations with different levels of developmental coupling.

### Do the costs of neural tissue select against concertedness when selection acts on a specific brain component?

**Fig. 3:**
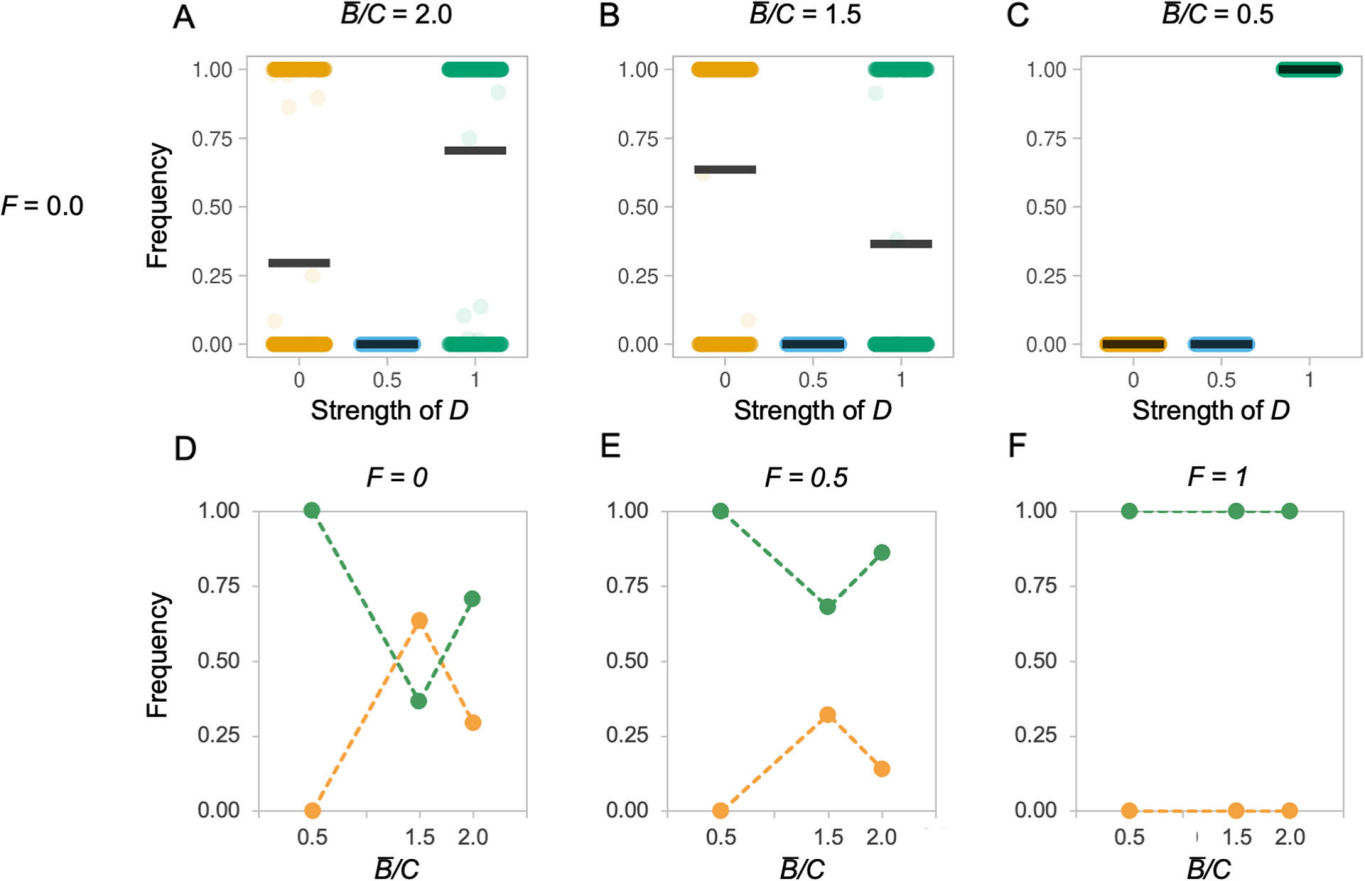
Selected examples of competition between evolving populations with different *D* values, showing the effects of varying the average benefit to cost ratio (*B̅/C*). **a**–**c** Each plot depicts the frequency of that *D* value relative to the total population (*y*-axis) as a function of developmental coupling *D* (*x*-axis) at the end of a 100-generation simulation run, compiled over 1000 simulation runs, with a mutation step size of 5%, and *F* = 0 to exemplify the effects of varying the *B̅/C* from 0 to 2. Populations are initialised such that there are 100 fully mosaic individuals (*D* = 0.0), 100 partially mosaic individuals (*D* = 0.5) and 100 concerted individuals (*D* = 1.0). Each data point is the outcome of one simulation run and the black bar indicates the mean of these runs. **d**, **e** Each plot depicts the average relative frequencies of *D = 0* (yellow) and *D = 1* (green) at the end of 1000 iterations of a 100-generation simulation, at three *B̅/C* ratios, across three *F* values representing low (**d**), moderate (**e**) and high (**f**) functional coupling. See Additional file 1: Figure S6-S8 for full results varying *B̅/C* and *F*, iteration numbers and mutation step size, and Additional file 1: Figure S9–11 for simulations in tailored environments that illustrate parameter effects

 In the preceding comparisons, the degree of mosaicism is also associated with the *B̅/C* ratio (*t* = 131.790, *p* < 0.001; Fig. 1d, Additional file 1: Figure S2), which interacts with *D* (*t* = − 90.970, *p* < 0.001) such that the degree of mosaicism tends to increase with *B̅/C* (Fig. 1d). We next repeated the simulations described in (ii) while varying the *B̅/C* ratio associated with additional brain tissue. The initial models were run with an average *B̅/C* ratio of 1.5 (Fig. 1, Additional file 1: Figure S6–8 s row) and were re-run with ratios of 2 and 0.5 (Fig. 3a–c, S6–8, first and third rows), simulating low and high costs of brain tissue. This revealed that relative tissue costs have a major effect on the success of populations with different *D* values (*t* = 12.116, *p* < 0.001). *B̅/C* interacts with *D* (*t* = − 13.885, *p* < 0.001) such that, for a given combination of *F* and *D*, a high *B̅/C* (=2) consistently increases the probability of success for the population with a high *D* value (Fig. 3a–c, Additional file 1: Figure S6-S8). In contrast, for a given combination of *F* and *D*, a moderate *B̅/C* (=1.5) consistently increases the probability of success for low D, mosaic models. However, when the *B̅/C* ratio is low (=0.5), the non-linear nature of the interaction between *C*, *F* and *D* is revealed, such that concertedness again becomes successful, even at low *F* values (Fig. 3d–f, Additional file 1: Figure S6-S8 third rows). In these competition experiments, mutation size had no effect on the outcome (*t* = 0.740, *p* = 0.459; compare Additional file 1: Figure S6 and S7). The non-linear success of low *D* values can be explained if developmental coupling facilitates rapid *decreases* in all brain regions when costs of brain tissue exceed the benefit, allowing a quick escape from a costly phenotype, or when the fitness of the whole system is dominated by a single component, such that increases in total brain size approximate the fitness benefit of increasing specific components.

**Fig. 4:**
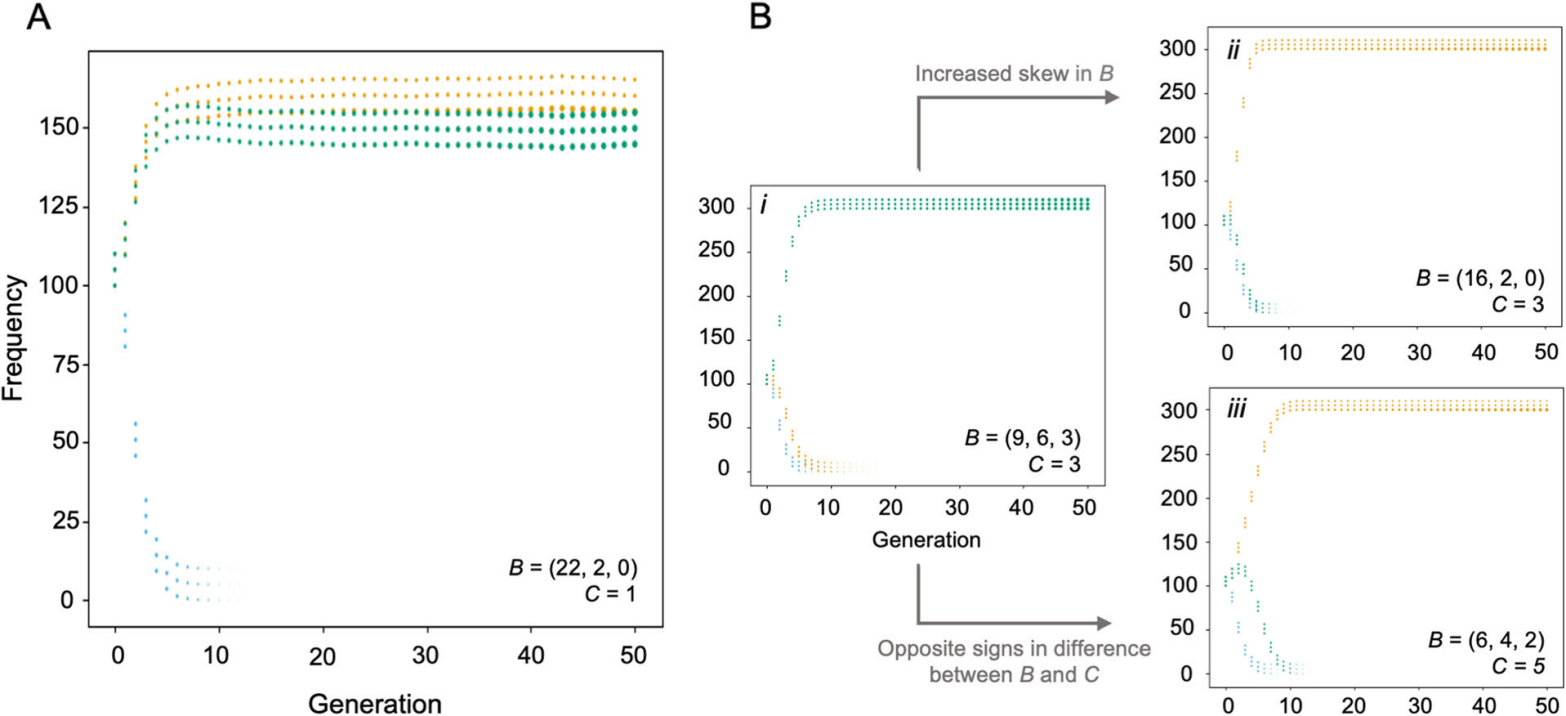
Selected examples of competition between evolving populations with different *D* values, showing the average frequency of each population during the first 50 generations of 1000 simulated runs, with ‘hand-crafted’ environments depicted in the figure to illustrate specific situations of interest. Each plot depicts the average relative size of the 3 components within the artificial brain, with *D* values colour coded (*D* = 0 in gold, *D* = 0.5 in blue, *D* = 1.0 in green). **a** When the benefits of each components are very strongly skewed, both *D* = 0 and *D* = 1 values can persist as each rapidly adjusts to approximate the optimal condition. **b** With moderate levels of variation in *B* across components, a *D* = 1 value spreads in the population (i); with increased skew in *B* across components, the probability of spreading switches to *D* = 0 (ii) unless skew is extreme in which case *D = 1* again becomes successful (**a**). Shifts in the probability of success for *D* = 1 are also associated with components having opposite signs in the *B̅/C* difference across components (ii)

To further illustrate these effects, we specified particular parameter comparisons that show how *B̅/C* interacts with changes in selection regimes such that the most successful *D* value switches based on changing the benefit-cost ratio (Fig. 4, Additional file 1: Figure S10-S12 rows) or variation of selection across components (Fig. 4, Additional file 1: Figure S10-S12 columns). In particular, we note that (i) simulations can maintain multiple populations with different *D* values where fitness is dominated by the contribution of one component (Fig. 4a); (ii) shifts in the probability of a population of a mosaic, low *D* population being successful are otherwise associated with increased variation in the *B* value among components (Fig. 4bii); and (iii) the relative success of a mosaic, low *D* population increases when one or two components provide a net benefit to size and other component(s) provide a net cost (Fig. 4biii), while populations with high *D* values are favoured when all components provide *either* a net benefit or a net cost (Fig. 4bi).

### Is developmental integration evolutionarily labile, and do functional dependencies select for developmental coupling?

**Fig. 5:**
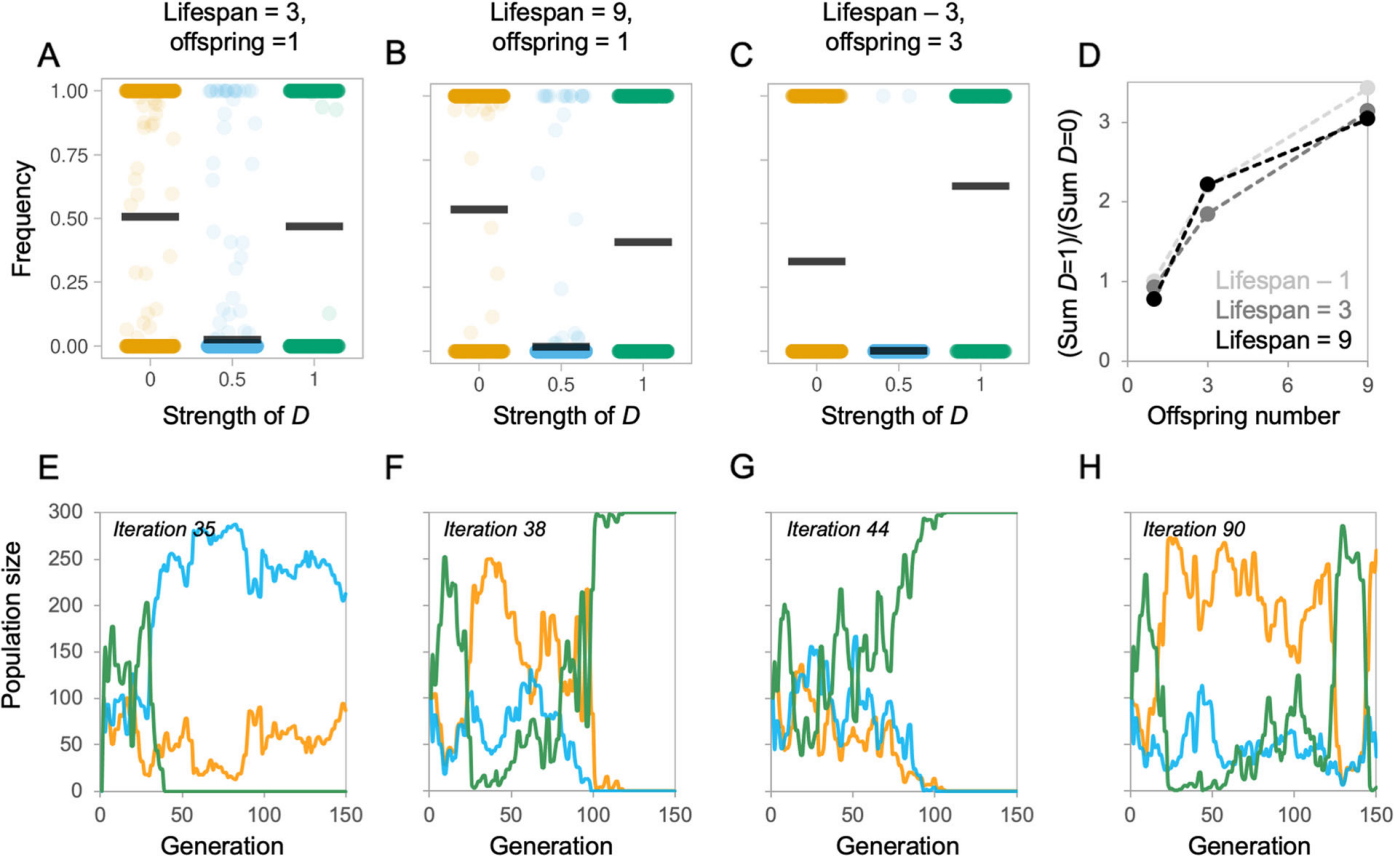
Selected examples of competition between populations with different *D* values in a randomly varying environment. **a**–**c** Each plot depicts the ratio of each population to the total population (*y*-axis) as a function of developmental coupling *D* (*x*-axis) at the end of a 150-generations, compiled over 1000 simulation iterations; every 2 generations, the environment was replaced, using a uniform random distribution for cost [0.5, 5], max average benefit [0.51, 105] and *F* functional coupling [0, 1]. Populations are initialized such that there are 100 fully mosaic individuals (*D* = 0.0), 100 partially mosaic individuals (*D* = 0.5) and 100 concerted individuals (*D* = 1.0). Simulations were performed varying two life history conditions: maximum lifespan, or the number of generations an individual persists alive, and offspring number, which are produced once every generation. Three combinations of lifespan and offspring are shown, for full comparisons see S13. **d** A summary of the ratio of concerted individuals (*D* = 1) to mosaic individuals (*D =* 0) at the end of each iteration of the 150 generation simulation, showing the effects of maximum lifespan and offspring number. **e**–**h** Selected, representative, individual simulations showing fluctuations in *D* value frequencies over generations. Colours indicate the *D* value, or *D* value, where yellow is *D* = 0, where blue is *D* = 0.5 and where green is *D* = 1. See Additional file 1: Figure S13-S16 for full results varying *B̅/C* and *F*, iteration numbers and mutation step size, and Additional file 1: S17 for effects of increasing the size of environmental fluctuations

We examined whether populations with alternative *D* values persist when the selection regime is temporally variable (due to randomised, independent changes in *B*, *C* and *F*). Under initial conditions, where offspring number was set to 1 and maximum age was set to 3, both *D* = 1 and *D* = 0 populations persist over 150 generations with roughly equal probabilities (Fig. 5a). Plotting the frequency of *D* values from individual simulations shows that the success of each population can fluctuate over time (Fig. 5e–g), with multiple populations persisting, on average, for 62 generations (Additional file 1: Figure S13A). This is substantially more than is found in simulations with fixed environments (Additional file 1: Figure S5) and is also reflected in the high average generation at extinction for each *D value* (*D =* 0, generation 91; *D =* 0.5, generation 57; *D =* 1, generation 98; Additional file 1: Figure S13). We subsequently varied maximum age and offspring number to explore how ‘slow’ (long lives, few offspring) or ‘fast’ (short lives, many offspring) life histories buffer the effects of environmental heterogeneity. This revealed that the main effect captured in the model was that of offspring number interacting with *D* (*t* = 43,622, *p* < 0.001), with higher offspring numbers increasing the probability of success of the concerted, high *D* value populations (Fig. 5a, c, d; Additional file 1: Figure S14-S16 columns). Increasing maximum lifespan had a significant but smaller effect in the opposite direction (*t* = − 4.349, *p* < 0.001; Fig. 5a, b, d; Additional file 1: Figure S14-S16 rows). Altering the amplitude of fluctuations in the environment also has a subtle effect on the probability of success of competing populations with different *D* values (*t* = − 8.986, *p* < 0.001), with more extreme conditions slightly increasing the success of concertedness (Additional file 1: Figure S18). In these competition experiments, mutation size again had no effect on the outcome (*t* = 1.000, *p* = 1.000; compare Additional file 1: Figure S14 and S15).

### Does stabilising selection or constraint on brain size lead to increased frequencies of mosaic evolution?

**Fig. 6.**
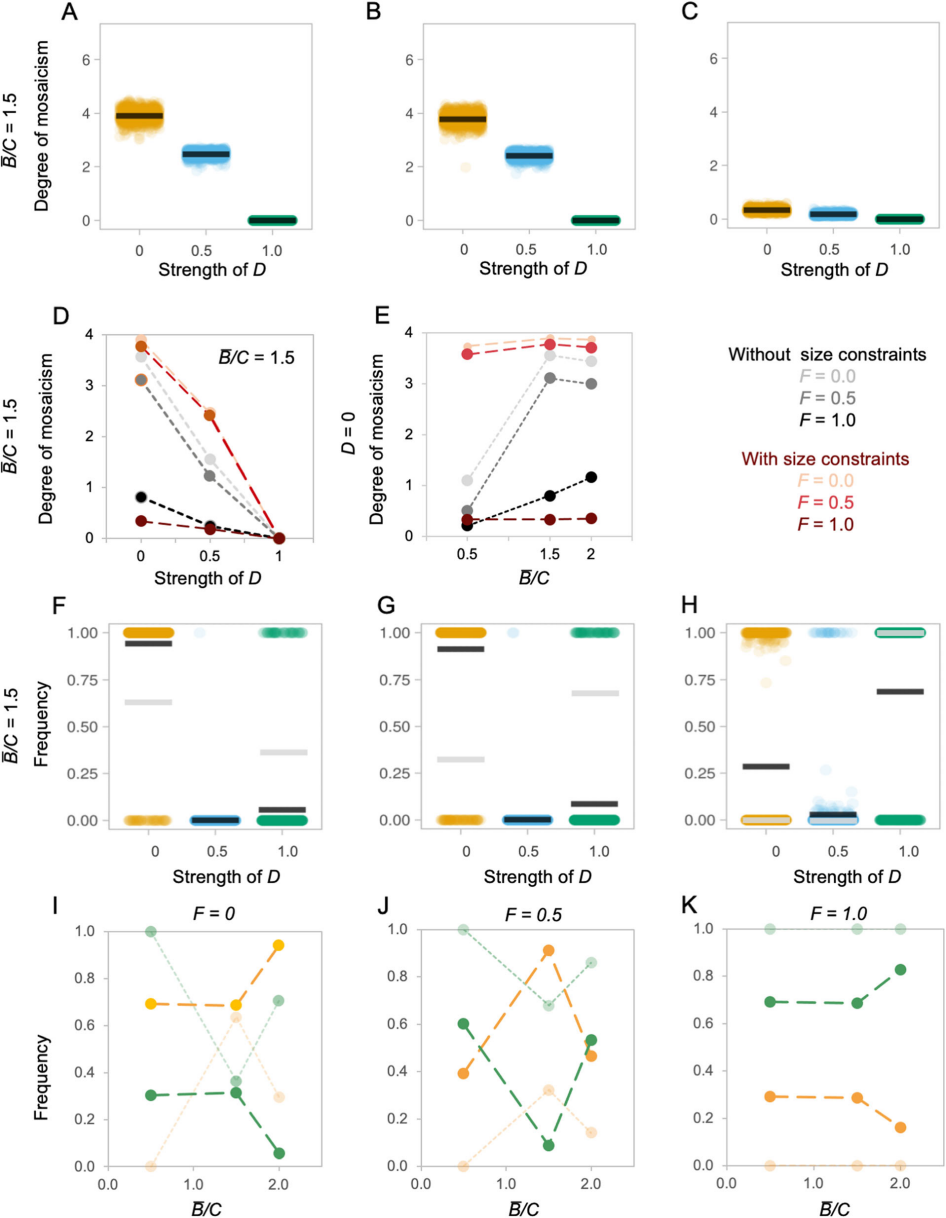
Mosaic and concerted evolution when brain size has upper and lower limits. **a**–**c** Evolution of ‘mosaicism’ under alternative conditions. **a**–**c** Each plot depicts the ‘degree of mosaicism’ (*y*-axis, defined as the natural log of the ratio between the largest brain component and the smallest brain component in each individual, averaged across the population) as a function of developmental coupling *D* (*x*-axis) under different environments, defined by their functional coupling *F*, with an average benefit to cost ratio (*B̅/C*) of 1.5 and a mutation step size of 5%. **d**, **e** Summary of the effects of varying *F* and *D*, and *F* and *B̅/C*, respectively, on the degree of mosaicism. **f**–**h** Selected examples of competition between evolving populations with different *D* values. Each plot depicts the frequency of that *D* value relative to the total population (*y*-axis) as a function of developmental coupling *D* (*x*-axis) under alternative environments defined by their functional coupling *F*, with a benefit to cost ratio (*B̅/C*) of 1.5 Each data point is the outcome of one simulation run and the black bar indicates the mean of these runs. For comparison, grey bars show the means from the same unbounded simulations in Fig. 2a–c. **i**–**k** Each plot depicts the average relative frequencies of *D = 0* (yellow) and *D = 1* (green) at three *B̅/C* ratios, across three *F* values representing low (**d**), moderate (**e**) and high (**f**) functional coupling. For comparison, results from the same unbounded simulations in Fig. 3d, e are shown in faded colours. See Additional file 1: Figure S20-S23 for further full results varying *B̅/C* and *F*

Imposing upper and lower bounds on the total size of the system has notable effects on the probability of mosaic and concerted evolution. Under these conditions, we again see that patterns of ‘concerted’ brain evolution can be caused by both developmental and functional coupling (Fig. 6a–c; Additional file 1: Figure S20). Mosaicism is more likely under all scenarios where *D* < 1 and *F* < 1 (*t* = 96.66, *p* < 0.001; Fig. 6d; Additional file 1: Figure S20). However, for a given *D* value, the degree of mosaicism is less impacted by variation in *B̅/C* than was observed in the boundless base model (Fig. 6d; Additional file 1: Figure S21). This can be explained by the dynamic nature of *C* when imposing upper and lower bounds on brain size. Regardless of the starting value, across iterations, *B̅/C* will tend to converge as brain size approaches its limits, which tends to happen well within the 100 generations of the simulations (Additional file 1: Figure S13). As a result, in some simulations, *D* = 1 populations initially increase in frequency until the population approaches the upper/lower boundary when the increasingly upscaled *C* results in the *D* = 0 population rising in frequency and becoming dominant (Additional file 1: Figure S22). In a competitive scenario, the relative frequency at which brains with low *D* values are favoured over brains with high *D* values also increases across the majority of parameter combinations (*t* = 36.88, *p* < 0.001; Fig. 6f–k), with a high *D* population being favoured in the majority of runs when *F* = 1, or for *F* = 0.5 when *B̅/C* is 0.5 or 2 (Additional file 1: Figure S23). Similar patterns are found in simulations across fluctuating environments with the frequency of *D* = 0 individuals in the final populations increasing across all runs (*t* = 24.59, *p* < 0.001; Fig. 6f–k).

## Discussion

The results of our agent-based simulations have several implications for studies using comparative volumetric data to interrogate mechanistic hypotheses of how brains evolve. First, they demonstrate that patterns of ‘concerted’ evolution (consistent allometric scaling of all brain components across macro-evolutionary time) should not be taken as evidence for developmental coupling, as concertedness can also evolve due to multiple mechanisms, including high functional interdependence in the total absence of developmental coupling. Second, depending on the context, both concerted and mosaic evolution can be adaptive, and the most probable route to adaptive brain evolution is strongly influenced by whether the change in selection regime is skewed towards one brain component or is evenly distributed across the whole brain. Third, probabilities of concerted or mosaic evolution are also dependent on what the relative costs and benefits of increased investment in brain tissue are. Fourth, our model shows that heterogeneity in selection regimes across time can result in both mechanisms being maintained in a population. Finally, we demonstrate that when upper and lower bounds are placed on brain size, the probability of mosaic evolution increases under most scenarios. Given these results, pluralism is a reasonable settlement. However, our results suggest ways of going beyond the apparent deadlock of empirical underdetermination and relative significance, as discussed in the introduction. It does this by opening and providing an initial exploration of new questions: Under what conditions do developmental coupling and uncoupling succeed? What causes switches between mosaic and concerted modes of evolution? And how can we empirically distinguish them? Our results further stress that mosaicism and concerted evolution are not competing models of brain evolution, but instead are reflections of evolutionary mechanisms that are jointly responsible for adaptive patterns of neural evolution.

### Concertedness is a phenotypic pattern devoid of mechanistic information

Our simulations clearly show that concerted brain evolution can occur under any level of developmental coupling, or *D* value (Fig. 1). This is consistent with classic quantitative genetics frameworks, which show that correlated evolution does not depend on strong genetic correlations among traits [35]. However, despite early arguments that this is the case [32, 33, 36], the distinction between concerted, or correlated, evolution and developmental constraints is often neglected in the debates surrounding the evolution of brain structure. In our model, the probability of concertedness predictably increases with *D*, but it also increases with *F*, even when *D* is 0. Why do high *F* values result in concerted evolution? If we assume that selection acts on specific brain components, and excess brain tissue is generally expensive, then selection to maintain functional relationships should be expected and would result in co-evolution among brain components. These formal results are consistent with empirical data. For example, across mammals, the major components of the visual processing pathway, including the peripheral visual system, lateral geniculate nucleus and visual cortex, tend to co-evolve with one another, as predicted by their functional integration [53–55]. Similarly, major components of the olfactory pathway, including the olfactory bulbs and olfactory cortex, also co-evolve [54, 56]. However, the visual and olfactory pathways show no consistent pattern of co-evolution between them [54]. Indeed, whether they co-vary negatively, positively or not at all can be explained by how diet and activity pattern interact to shape foraging behaviour [54]. In addition, major brain structures connected by long-range axons, such as the neocortex and cerebellum, also tend to co-evolve independently of total brain size, while also showing evidence of temporally transient independent change [57–60]. These examples illustrate the effects of functional integration on co-evolution among brain components which are consistent with our model outputs. Finally, our model demonstrates an interaction between *D* and *F*, which may suggest that a pattern of concertedness driven by *F* could select for, or against, developmental integration. Regardless, our results demonstrate that concerted patterns of brain evolution provide no real evidence either for or against the prevalence of developmental coupling.

### Both concerted and mosaic evolution can be adaptive, depending on the costs and benefits of brain tissue, and the complexity of the mutation landscape

Our simulations suggest that the cost of excess brain tissue and the distribution of selection across networks of brain components play critical roles in determining how brains evolve. When the strength and direction of selection is skewed towards one brain component, the probability that a population with mosaic brains, or low *D* values, will be successful is increased. However, when the relative costs of neural tissue are either very low or high, the balance can switch to favour high *D* values. This likely reflects the ‘speed’ at which the two populations can respond to selection. With only one mutational mechanism, it is potentially more likely for a developmentally coupled brain to evolve towards a new ‘adaptive peak’ than it is for a mosaic brain. This is because the mutational landscape for a mosaic brain is more complex, and the probability of hitting the optimal mutational path is reduced by a greater number of potential mutational outcomes.

At face value, our simulations therefore support previous arguments that ‘… a coordinated enlargement of many independent components of one functional system without enlargement of the rest of the brain may be more difficult, as its probability would be the vanishingly small product of the probability of each component enlarged individually’ (2, p. 1583). However, if the costs of brain components are unequal, as may be expected if energetic consumption scales with neuron number [61] and neuron density varies between components [62], then this effect would be dampened according to the distribution of costs and benefits across the whole system. Hence, an uneven distribution of neural costs would likely increase the probability of mosaicism. Evidence that this effect occurs in nature may be provided by recent data showing life history traits constrain specific brain regions independently of overall brain size [63].

In addition, when we impose an upper and lower bound on brain size, to reflect scenarios where the total size of the system is under constraint, for example due to brain/body allometry, we further see the importance of considering the costs of neural tissue. Here, the cost of brain expansion increases as the system approaches either boundary, such that the option to inflate brain size as a whole becomes increasingly problematic. Under these conditions, the probability of mosaicism increases as it becomes more energetically conservative, and in some parameter spaces, individuals may have to reallocate energetic investment from one brain region to another, potentially resulting in investment trade-offs. We also note that, although we present the results of one way in which costs may scale under brain size constraints, the results of alternative cost scaling relationships can be clearly predicted from formula 2; with altered settings for upper and lower bounds, the effects would ‘kick in’ at earlier or later points, and with different *C* exponents, the effects would ‘kick in’ with faster or slower rates.

While accurately assessing the fitness costs and benefits of brain tissue remains challenging, we suggest some tentative predictions based on these interpretations. First, we expect mosaicism to be less likely during transitions to high-quality diets, as the cost of neural tissue relative to the overall energy budget may be reduced. Second, under sudden periods of energy resource limitation, brains should shrink, at least initially, in a concerted manner, before approaching a lower bound for some structures when mosaicism will kick in to adaptively restructure neural investment. Third, mosaicism may be more likely when energy intake is relatively constant, but selection favours changes in neural investment which involve energetic trade-offs. This may be common during periods of ecological change that are not associated with changes in body size (and by proxy energetic intake), or in taxa that are size limited. It is tempting to interpret some notable patterns of brain evolution in this context. For example, within hominin evolution, human brains are largely structured in a way that is consistent with neural scaling across hominoids despite its massive increase in size [64, 65]. Given that some authors have argued brain expansion was facilitated by dietary shifts [66–68], this could have provided the conditions for a concerted pattern of brain expansion. In contrast, the brain of the diminutive hominin *H. floresiensis* is likely to have evolved under considerable size constraints and other authors have suggested cognitive evolution in this lineage was facilitated by a distinct pattern of change in brain morphology, perhaps reflecting a response to selection under size constraints [69]. Of course, further experimentation with amenable systems will be necessary to test the predictions of our model.

### Partially mosaic brains and the maintenance of diversity

It may be surprising that partially mosaic individuals, which are seemingly a compromise-state, generally have low probability of success in our simulations. This can again be explained by the ‘cost’ of mutation. In short, partially mosaic individuals incur the highest cost of mutational complexity. If the direction of mutation is random, in a given generation, the partially mosaic brain simply has lower odds of increasing its fitness because mutations affecting the whole brain and region-specific size may often be in conflict. The relative lack of genetic variation simultaneously contributing to both whole brain and region-specific size in natural populations [5, 8, 39] is in keeping with this conclusion. However, how mutational effects interact during brain development requires further investigation.

However, our simulations across temporally variable selection regimes indicate that populations with alternative *D* values can co-persist within a single population for many generations. This provides an alternative route to mixed temporal patterns of concerted and mosaic evolution within a single population. If we view *D* values in our model as alternative genotypes, this implies that genetic variation affecting specific components and genetic variation affecting total brain size may both either persist at low levels in natural populations or periodically arise de novo and spread through a population. Regardless, this would enable selection to fluctuate between favouring mosaic and concerted mechanisms, permitting both adaptive restructuring and adaptive conservation of brain structure, without invoking genetic mechanisms that partly link brain structure size and whole brain size.

### Reconciling mosaic and concerted views

Our simulation results suggest a way of reconciling mosaic and concerted views of brain evolution. Developmentally coupled brains evolve in scenarios involving some combination of tissue costs being evenly distributed, and an extreme and variable fitness landscape, while mosaic brains are the result of environmental stability coupled with differentiated selection among components, and/or strong constraints on brain size. The quick evolutionary response enabled by developmentally coupled brain evolution makes it ideal for circumstances where getting it ‘approximately correct’ quickly is advantageous, while mosaic evolution is favoured when accuracy trumps speed. This is also tied to life history and environmental heterogeneity. For example, large offspring number, which increases short-term competition, favours the fast response provided by developmental coupling, while lower competition allows mosaic populations to persist, find the optimum brain structure and out-compete developmentally coupled individuals.

The contrasting benefits of concerted and mosaic evolution bring us back to the initial major division between the two polarised hypotheses [1, 2, 13–15, 17] which continues to be widely reflected in the literature (e.g. [4–12]); where developmental coupling does occur, is it a constraint, or has it evolved and is therefore *evolvable*? Our simulations support developmental coupling as a scenario-dependent adaptive mechanism, rather than a constraint. First, our simulations show that the fate of the two opposing mechanisms can vary through time. In nature, this would be reflected in the formation and breaking of genetic correlations between traits. Second, the general absence of genetic correlations observed in quantitative genetics studies is reconciled with concertedness via developmental coupling by invoking the importance of de novo mutation, but our simulations suggest that the probability that these mutations will spread to fixation depends on the environmental context. By showing that the distribution and size of costs and benefits are critical in determining the outcome of competing mechanisms, our model supports a view whereby global selection regimes determine the constraints acting on brain evolution, not the developmental program. Two key conclusions from this work are therefore (i) patterns of concertedness should not be equated with developmental coupling and (ii) developmental coupling should not be equated to developmental constraint.

## Conclusion

In sum, our agent-based simulations of alternative views of brain evolution provide a number of informative predictions that should refresh our view of this long running debate. First, we show concertedness is an outcome, not a mechanism, and on its own does not provide evidence for developmental coupling or constraint. Second, we demonstrate that selection regime and structural interdependencies are critical to the outcome of competing mechanisms. Third, we argue that developmentally coupled and uncoupled brain architectures can both be adaptive, but we contest the assumption that developmental coupling is necessarily a persistent evolutionary constraint. Finally, our model provides a way to integrate patterns of brain evolution, life history and environmental heterogeneity. We of course acknowledge that our model is a simplification of a highly complex biological phenomenon. While the assumptions we make in constructing the model are intended to help us clearly explore previous verbal arguments, several extensions are possible. These include the addition of heterogeneous distributions of energetic costs, costs of functional integration to mirror energetic costs of long-range axons, and a degree of plasticity in brain structure and size that would reflect the plasticity of neural systems in response to their social and physical environment. Nevertheless, we hope the general approach taken can trigger greater formalisation of evolutionary hypotheses in this field, and work that further refines our computational models.

## Methods

To explore the interplay between developmental and functional coupling on the evolution of brain structure, we devised a model that simulates the evolution of a population of individuals in an environment, where fitness is determined by the size and costs of brain components.

### Definitions

We employ terminology from the evolutionary neurobiology literature, but the debate could also be understood in terms of principles derived from quantitative genetics. ‘Concerted evolution’ is the result of correlated evolution among all brain components, which can be caused by two main processes. The first, referred to as ‘developmental coupling’, occurs through genetic correlations that act through development, such that variation at a particular locus affects the development of multiple, or all, brain components. ‘Developmental de-coupling’ refers to the breakdown, or absence, of these genetic correlations. The second, referred to as ‘functional coupling’, instead occurs through correlated selection pressures, which either arise due to components contributing to shared behavioural or computational functions, or the environment selecting on multiple aspects of brain structure (e.g. dark environments selecting for increased olfactory processing and against visual processing; in this case, the selection correlation is negative). Both correlated selection and genetic correlations can lead to co-evolution between traits [35], and our model is designed to explore this in the context of brain evolution. However, we note it is likely applicable to other anatomies.

### Base model components

The model is characterised by three components: the population of individuals, the environment and an evolutionary algorithm. We provide a simplified description of the model in Additional file 1: Figure S1.

Each individual is characterised by a number (*N*) of *brain components*, each component having a specific *size* (*S*_*i,j*_), where *i* denotes the individual and *j* denotes the *j*th of *N* components. For example, among individuals with three brain components (*N* = 3), the brain of ‘Individual A’ could have a first component of size *S*_*A,1*_ = 5, a second component of size *S*_*A,2*_ = 7 and a third component of size *S*_*A,3*_ = 2, which we denote as *S*_*A*_ = (5, 7, 2). A second individual, ‘Individual B’, might have component brain sizes of 3, 3 and 1, respectively, denoted *S*_*B*_ = (3, 3, 1). In this case, the total size of Individual A’s brain would be 14, while the size of Individual B’s brain would be 7. Our model assumes that all brain components have the same fitness cost per unit size (see below), so it is total brain size that determines the evolutionary cost of an individual’s brain, while the size of individual components contributes differently to fitness benefits. Whether evolution is mosaic or concerted depends upon the factors influencing changes in brain component sizes over time. The sizes of brain components are allowed to vary, through mutation, and this variation is influenced by *developmental coupling* (*D*), which takes values between 0.0 (no developmental coupling, i.e. a fully ‘mosaic’ brain) and 1.0 (complete developmental coupling, i.e. a brain structure fully determined by total brain size). *D* is analogous to the strength of genetic correlations between components. For example, a *D* of 0.5 would indicate that 50% of the variation in each component is determined by variation in total brain size, and 50% of the variation in each brain component is independent of variation in both total brain size and other components. When a mutation event occurs, the program generates *N* + 1 random mutation factors (*m*_*j*_), for example between 0.5 and 1.5 for a 50% mutation step size, where there is one factor for the whole brain (*m*_*0*_) and one for each component (*m*_*1*_ to *m*_*N*_), each brain component is then scaled by these mutation factors, with the variation in mutations affecting particular brain components being flattened depending on *D’s* value. For example, when *D* is 0, *m*_*0*_ for the total brain size mutation will be multiplied by 0 but other mutation factors will vary independently according to a scaling factor (1 – *D*, i.e. unscaled when *D* is 0), whereas when *D* is 1 all mutation factors for individual brain components will be multiplied by 0 and only the mutation factor for total brain size will persist. Models with intermediate values of *D* fall between these extremes. This is determined according to the following formula:

1 $$ {S}_{i,j(new)}=\left[\left(D\times {m}_0\right)+\left(\left(1-D\right)\times {m}_j\right)\right]\times {S}_{i,j(old)};j\in \left\{1,\dots, N\right\} $$

The *Environment* is characterised by three factors. First, an environment is characterised by *Functional Coupling* (*F*), between 0.0 (no functional links between two brain components) and 1.0 (complete functional interdependence between two brain components), which determines how similar brain component benefits are to each other (for *F* = 1, all benefits are identical) — see below for implementation details. Second, a set of *benefits* (*B*_*j*_), one for each of the *N* brain components, which represents the fitness contribution of each size unit an individual gains from that particular brain component, at a particular size. For example, in an environment with benefits *B* = (1, 2, 3), Individual A, from above, will have total fitness contributions from brain component sizes of 5 × 1 + 7 × 2 + 2 × 3 = 25, while Individual B will have total fitness benefits of 3 × 1 + 3 × 2 + 1 × 3 = 12. This is implemented by varying *B*_*max*_, the highest possible benefit a unit of size could give, with the benefit per component size *B*_*j*_ given as a random fraction (uniformly sampled from the range 0 to 1) of this maximum benefit. Where *F =* 1, the generated fraction is the same for all components; if *F* = 0, then the benefit per component is independent (i.e. three generated fractions), and for any intermediate value of *F*, the benefit provided by each component is determined by contributions from both the cross-brain fraction and the per-component fractions, scaled so that the sum of fractions per component never exceed 1. With higher *F* values, the individual component benefits are constrained to be more similar. The average benefit per component is always half the maximum benefit permitted, and the degree of functional coupling (*F*) determines how correlated they are. Third, the environment imposes a fitness *cost* (*C*) per size unit of the brain, which is uniform for all brain components based on the assumption that there is a linear ‘per neuron’ energetic cost [61], and that units of ‘size’ in the model are analogous to neuron number. More specifically, the units of ‘size’ in the model are analogous to the ratio between the neuron number of the current organism and the neuron number for the common ancestor, with the common ancestor starting with equal sized brain components, *S*_*0*_ *=* (1,1,1), in all our simulations. Total fitness for an individual *i* with brain component sizes *S*_*i,j*_ in an environment defined by benefits *B*_*j*_ and cost *C* is thus given by $\sum \limits_j\left({S}_{i,j}\times {B}_j\right)-\left(C\times \sum \limits_j{S}_{i,j}\right)$. For example, if the environment described above had a cost *C* = 1, Individual A will experience a fitness cost of 14, giving a total fitness of 25–14 = 11, while Individual B will experience a fitness cost of 7, giving a total fitness of 5. To visualise the effects of varying *C* and *B*_*max*_, we measure the ratio of the average *B* across components (annotated *B̅*), to *C*.

The *Evolutionary Process* progresses through the following steps:

1. Determine the number of ‘offspring’ for each individual in a population, and the age of all individuals, measured in the number of generations (these are identical across the population).
2. Initialise an environment and a population of individuals with identical, uniform brain component sizes (*S*_*i,j*_ = 1).
3. For a given number of simulation steps
  - Generate offspring for all individuals
  - Mutate all offspring
  - Increase the age of all individuals
  - Remove individuals whose age exceeds the maximum age
  - Rank individuals according to their total fitness in the environment (calculated as described above)
  - Remove the lowest ranking individuals until the population size returns to the origin size (i.e. population size is stable over time)

Given the above model parameters, we can examine the effects of developmental coupling (*D*) and functional coupling (*F*) on the evolution of brain component sizes. We can also explore the evolution of brain structure in populations of individuals with an intermediate value of *D* (unless specified otherwise, we use *D =* 0.5), which are neither fully concerted nor fully mosaic. We call these ‘partially mosaic’ individuals, where some of the mutations affect total brain size, scaling each component equally, and some affect each component independently, as given by Eq. (1) above. We can then assess which mechanism, for example, a fully mosaic brain (*D* = 0), a fully concerted brain (*D* = 1) or a partially mosaic brain (*D* = 0.5), is most successful in different scenarios by measuring the frequency of individuals in a population with that *D* value, as a proportion of total population size, after *n* generations. The model was initially implemented with no upper ceiling on overall brain size, in which case it most accurately simulates periods reflecting directional increases/decreases in brain components, and by extension brain size, which is a common but not universal trend [70, 71]. This means that under static selection regimes the base model can lead to continuous directional changes in total brain size. We also allow the environment to change randomly over the course of a simulation to examine how temporal heterogeneity in selection regimes affects the long-term success of alternative brain models.

### Introducing constraints on total brain size

As described above, the initial model imposes no upper or lower limit on total brain size. While this will reflect periods of directional changes in the brain, or brain component, size [70, 71], the close correlation between brain and body size, which may evolve under contrasting selection pressures [35], may impose limitations on how brains respond to selection that is not captured in the base model. However, these upper and lower boundaries can be envisaged in terms of non-linear relationships between the size of brain components and their costs. As brain size approaches an upper/lower ceiling, the relative cost of increasing/decreasing each component is likely increased, resulting in increased disparity of *B̅/C* ratios for each component when selection is favouring increases in particular brain regions. To compare how such boundaries may impact the probability of different outcomes for *D* values, we implemented an extension to the model in which *C* is scaled according to its distance from the brain size of the common ancestor, with costs becoming increasingly prohibitive near an upper or lower boundary, set as 1.5 and 0.5 times the starting combined size of all three components, respectively. This scaling factor was implemented as:

2 $$ {C}_i=C\times 0.25\times {\left(\frac{\sum_j{S}_{0,j}}{\sum_j{S}_{i,j}}+\frac{\sum_j{S}_{i,j}}{\sum_j{S}_{0,j}}\right)}^2 $$

where *C* is the environmental-determined base cost, ∑_*j*_*S*_*i*, *j*_ is the total size of all brain components for the current individual and ∑_*j*_*S*_0, *j*_ is the total brain size of the common ancestor. Under these conditions, the model aims to examine situations where variation in total brain size is under stabilising selection or some form of constraint.

### Comparing effects of parameter variation on probabilities of mosaicism

Using the base model, we first conducted a series of simulations to explore four key questions identified in the introduction:

1. What mechanisms can produce concerted evolution? Here, we fixed *C* to equal 1 and fix *B*_*max*_ to be 1, 2 or 4, giving a range of average *B̅/C* conditions, while varying *D* and *F*. We then ran simulations to examine the degree of mosaicism observed under high, moderate and low levels of *F* and *D.*
2. Can both mechanisms be adaptive? Here, we repeat the comparisons above, but in competitive environments to examine the probability of obtaining coordinated changes in brain components under high, moderate and low levels of *F.*
3. Do the costs of neural tissue select against concertedness when selection acts on specific brain components? i.e. How does variation in fitness contributions from different components affect the way brains respond to selection? This can be addressed in two ways: first, by varying *F*, which determines how correlated *B* values of each structure are, or by varying *B*_*max*_, to alter the *B̅/C* ratio. Here, our aim was to test whether different levels of variation in the fitness contribution of additional brain tissue alter the probability of obtaining a mosaic or concerted brain.
4. Is developmental integration evolutionary labile? i.e. in a fluctuating environment do strong developmental constraints evolve and/or collapse? In this comparison, we took a different approach. We introduced a starting population of brains with a range of component sizes and *D*. We then allowed *C* and *B*_*max*_ to vary randomly every 2 generations to test what combination of factors persist over time. Here, costs were sampled uniformly in the range 0.5–5, and *B*_*max*_ was sampled uniformly in the range 1–10. As a result, the average benefit (*B̅*) is in the range 0.5–5, which is the same range as the cost, but the actual *B̅/C ratio* will vary widely between generations. *F* was also sampled uniformly in the range 0–1. We subsequently explored how varying key life history traits (numbers of offspring and maximum age) might buffer the effects of random environmental fluctuations.
5. How do constraints on brain size interact with the probability of mosaicism? Finally, we subsequently examined how imposing upper and lower bounds on total brain size impact the probability of obtaining a mosaic or concerted brain under each of the conditions described above.

In all case, the simulations were run over 100 generations, with 1000 iterations, a fixed population size of 300 individuals, initiated with 100 individuals per *D* value. In the main text, we present results of simulations with a mutation step size of 5%, but runs using the base model were also repeated with a larger mutation step size of 50% to examine how effects were influenced by mutation size (full results are presented in the Supplementary Information). To explore the early stages of the simulations, we also present results from a subset of simulations with 10 generations for these base comparisons. In experiments 2 and 3, simulations were run with an initial population containing equal numbers of individuals with different *D* values (*D* = 0, 0.5 or 1), which were then evolved under different environment conditions (determined by *F* and the ratio of benefits to cost). In experiment 4, environmental conditions were randomly varied every 2 generations for 150 generations. When imposing the upper and lower bounds on brain size, we repeated experiments 1–4, as described above, with a mutation step size of 5%. The ‘success’ of a *D* value was determined by the proportion of individuals in a population with that *D* value at the end of the simulation run. The full output of all models are summarised in Additional file 1: Figure S2-S23. The frequency of *D* values was compared using generalised linear models and the glm() function in R [72] across batches of nine, 1000-iteration simulations where *F*, *D* and *B*_*max*_ *and/or C* varied (experiments 1–3, see for example Additional file 1: Figure S2,S6), or where *F*, *D*, maximum lifespan and offspring number varied (experiment 4, see, for example Additional file 1: Figure S14) (all experiments total *n =* 27,000 iterations). We estimated the effects of all parameters and interactions where indicated, with a Gaussian distribution when comparing ‘degrees of mosaicism’ (defined as the natural log of the ratio between the largest brain component and the smallest brain component in each individual, averaged across the population) and a quasibinomial distribution when comparing proportional frequencies.

The code files in which the model is implemented are openly available for readers to implement additional parameter settings, or to extend the model, and can be accessed from github.com/shaharavin/BrainEvolutionSimulator [73]. Biplots were made using PlotsOfData [74]. Many simulations produce a bimodal distribution of frequencies, we therefore display the mean of these iterations solely to illustrate the skew in the outcome.

## Supplementary Information

**Additional file 1: Figures S1-S23. Figure S1.** A simplified, pictorial depiction of the model. Pages 4–5. **Figure S2.** Evolution of ‘mosaicism’ under alternative conditions, full comparisons, run with 5% mutation size and 100 generations (sister to Fig. 1). Page 6. **Figure S3.** Evolution of ‘mosaicism’ under alternative conditions, full comparisons, run with 50% mutation size and 100 generations (extension of Fig. 1). Page 7. **Figure S4.** Evolution of ‘mosaicism’ under alternative conditions, full comparisons, run with 50% mutation size and 10 generations (extension of Fig. 1). Page 8. **Figure S5.** Generation number at convergence during simulations of competition between evolving populations with different *D* values under alternative conditions (companion to Fig. 2). Page 9. **Figure S6.** Competition between evolving populations with different *D* values under alternative conditions, full comparisons, run with 5% mutation size and 100 generations (sister to Fig. 2). Page 10. **Figure S7.** Competition between evolving populations with different *D* values under alternative conditions, full comparisons, run with 50% mutation size and 100 generations (extension of Figs. 2 and 3). Page 11. **Figure S8.** Competition between evolving populations with different *D* values under alternative conditions, full comparisons, run with 50% mutation size and 10 generations (extension of Figs. 2 and 3). Page 12. **Figure S9.** Competition between evolving populations with different *D* values under tailored environmental conditions, run with 5% mutation size, showing the average size of each brain component and the frequency of competing *D* values (extension of Figs. 2 and 3). Page 13. **Figure S10.** Competition between evolving populations with different *D* values under tailored environmental conditions, full comparisons, run with 5% mutation size and 100 generations (extension of Figs. 2 and 3). Page 14. **Figure S11.** Competition between evolving populations with different *D* values under tailored environmental conditions, full comparisons, run with 50% mutation size and 100 generations (extension of Figs. 2 and 3). Page 15. **Figure S12.** Competition between evolving populations with different *D* values under tailored environmental conditions, full comparisons, run with 50% mutation size and 10 generations (extension of Figs. 2 and 3). Page 16. **Figure S13.** Conditions at convergence of simulations of competition between evolving populations with different *D* values in a randomly varying environment, under different life history conditions (companion to Fig. 2). Page 17. **Figure S14.** Competition between evolving populations with different *D* values in a varying environment, under different life history conditions, full comparisons, run with 5% mutation size and 100 generations (sister to Fig. 4). Page 18. **Figure S15.** Competition between evolving populations with different *D* values in a varying environment, under different life history conditions, full comparisons, run with 50% mutation size and 100 generations (extension of Fig. 4). Page 19. **Figure S16.** Competition between evolving populations with different *D* values in a varying environment, under different life history conditions, full comparisons, run with 50% mutation size and 10 generations (extension of Fig. 4). Page 20. **Figure S17.** Selected, representative, individual simulations showing fluctuations in population frequencies over 100 generations for a 5% mutation size (A-D) or a 50% mutation size (E-H) (extension of Fig. 4). Page 21. **Figure S18.** Subtle effects of the size of environmental fluctuations on competition between evolving populations with different *D* values in a varying environment (extension of Figure S16). Page 22. **Figure S19.** Relationship between population frequencies between partially mosaic brains (*D* = 0.5) and fully mosaic (*D* = 0), or concerted brains (*D* = 1) from simulations in varying environmental conditions and a 5% mutation size (A) or 50% mutation size (B) (extension of Fig. 4). Page 23. **Figure S20.** Evolution of ‘mosaicism’ under alternative conditions with upper and lower bounds on brain size, full comparisons, run with 5% mutation size and 100 generations (extension of Fig. 6). Page 24. **Figure S21.** Competition between evolving populations with different *D* values under alternative conditions with upper and lower bounds with upper and lower bounds on brain size, full comparisons, run with 5% mutation size and 100 generations (extension of Fig. 6). Page 25. **Figure S22.** Selected, representative, individual simulations showing fluctuations in population frequencies over 50 generations for a 5% mutation size, with upper and lower bounds with upper and lower bounds on brain size (extension of Fig. 6). Page 26. **Figure S23.** Conditions at convergence of simulations of competition between evolving populations with different *D* values in a randomly varying environment, with upper and lower bounds on brain size, under different life history conditions (extension of Fig. 6). Page 27.

## Data Availability

The code generated during the current study are available at github.com/shaharavin/BrainEvolutionSimulator [73].
